# INTERVENTIONS FOR COGNITIVE FRAILTY: DELPHI CONSENSUS WITH RESEARCHERS, CLINICIANS, AND EXPERTS BY EXPERIENCE

**DOI:** 10.1093/geroni/igae098.1184

**Published:** 2024-12-31

**Authors:** Carol Holland, Nikolette Dravecz, Susan Broughton, Sally Fowler Davis, Mandy Dixon

**Affiliations:** Lancaster University, Lancaster, England, United Kingdom; Lancaster University, Lancaster, England, United Kingdom; Lancaster University, Lancaster, England, United Kingdom; Anglia Ruskin University, Cambridge, England, United Kingdom; The Northern Health Science Alliance Ltd, Manchester, England, United Kingdom

## Abstract

The conjunction of physical frailty and cognitive impairment without dementia is described as Cognitive Frailty (CF). Indications that CF is potentially reversible has led to proposals that addressing risk factors, symptoms or mechanisms of CF would be appropriate targets for interventions for prevention, delay or reversal. However, no study has brought experts together across sectors to determine targeted or structures of interventions, and most resources on interventions are from the perspective of academic or clinical researchers only. We conducted an international Delphi consensus study bringing together experts from academic and clinical research, lay people with lived experience of CF, informal carers, and professional care practitioners/clinicians. Three rounds of the Delphi study were held to discern which factors were agreed upon by the whole sample and on which did people with differing expertise have differing views. A scoping review and Round 1 was used to gather initial statements. In Round 2, 56 people responded to statements and open text boxes, with 7 lab based researchers, 27 researchers working with people, 14 people with lived experience or informal family carers, and 10 professional carers/clinicians. Analysis of quantitative data gave 74 statements on which there was at least 70% agreement and qualitative data gave 24 statements, which were then combined to give 90 statements for Round 3, to which 38 original participants responded. Consensus was found for 88 of the statements. Differences between the groups were observed at both stages. Outcomes can be used to feed into co-creation of interventions for cognitive frailty.

